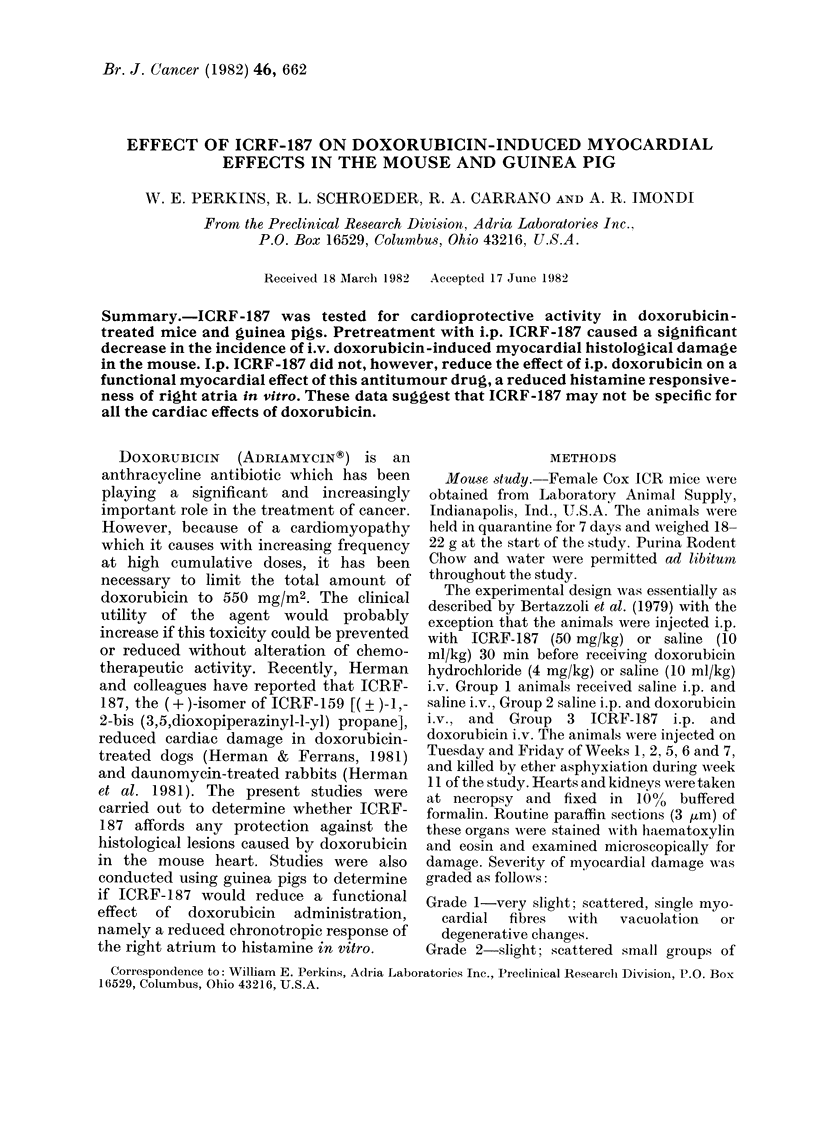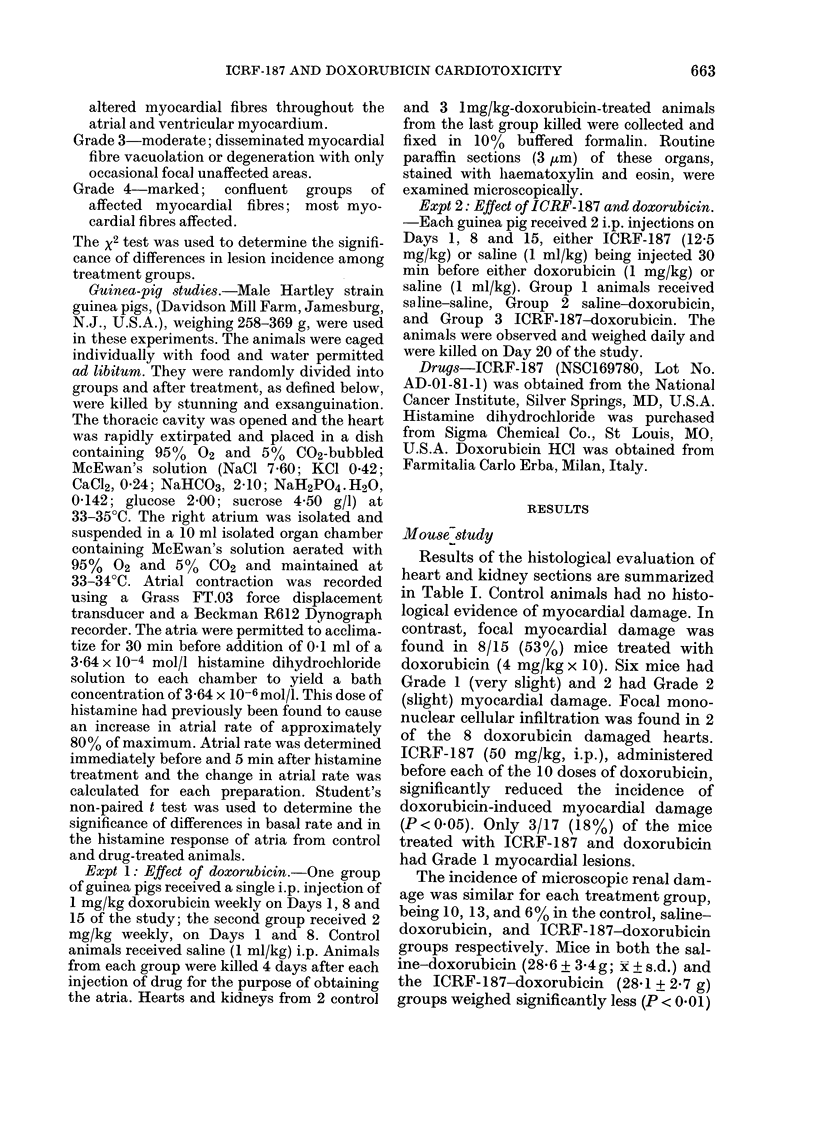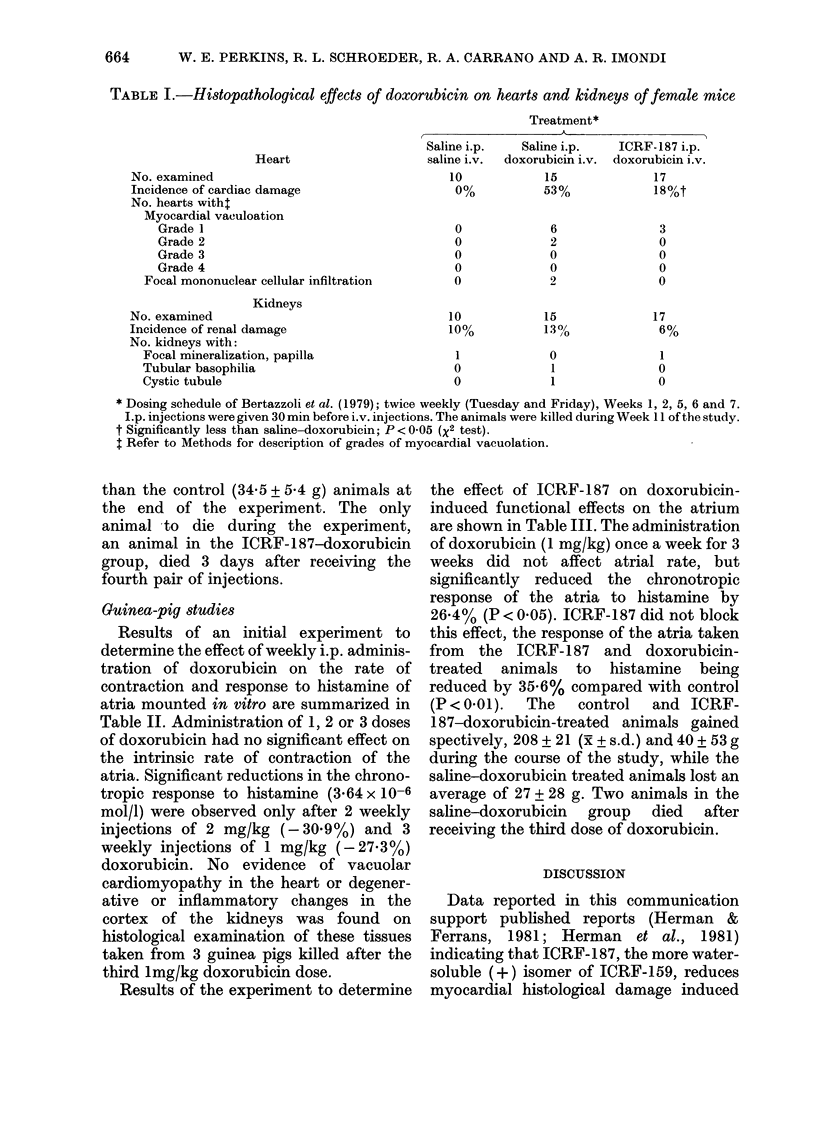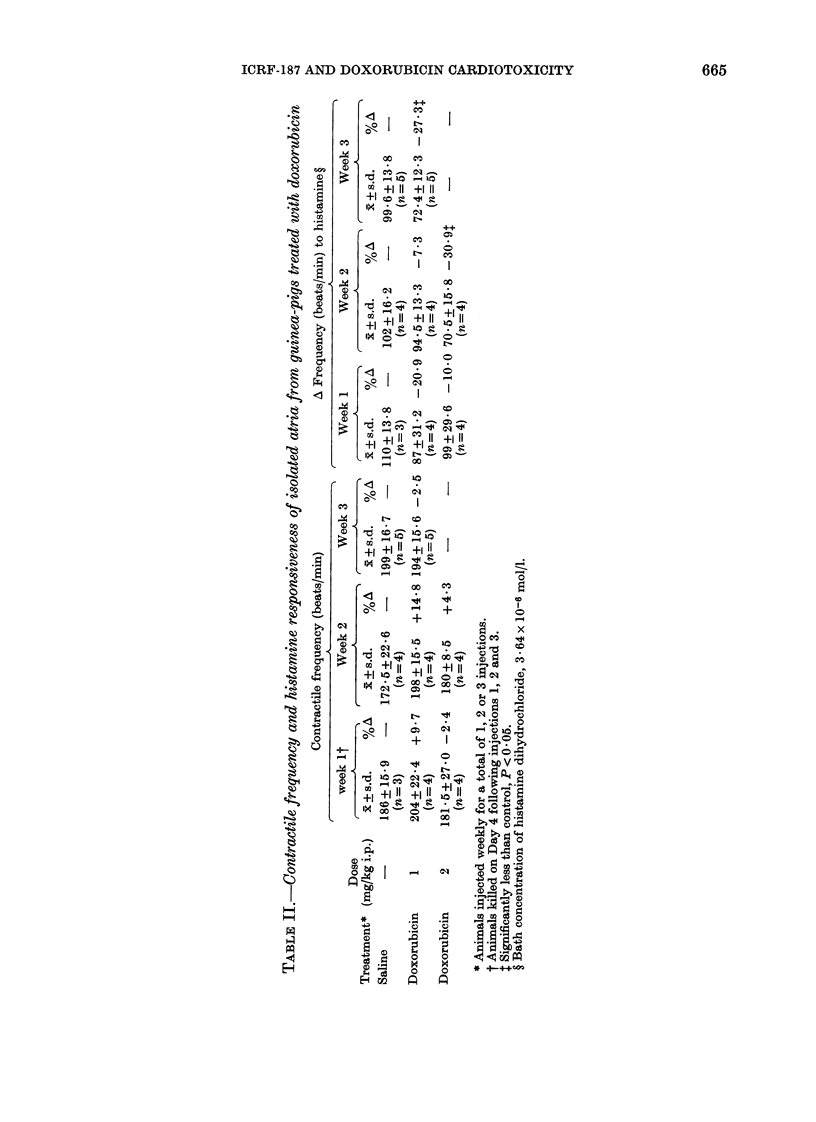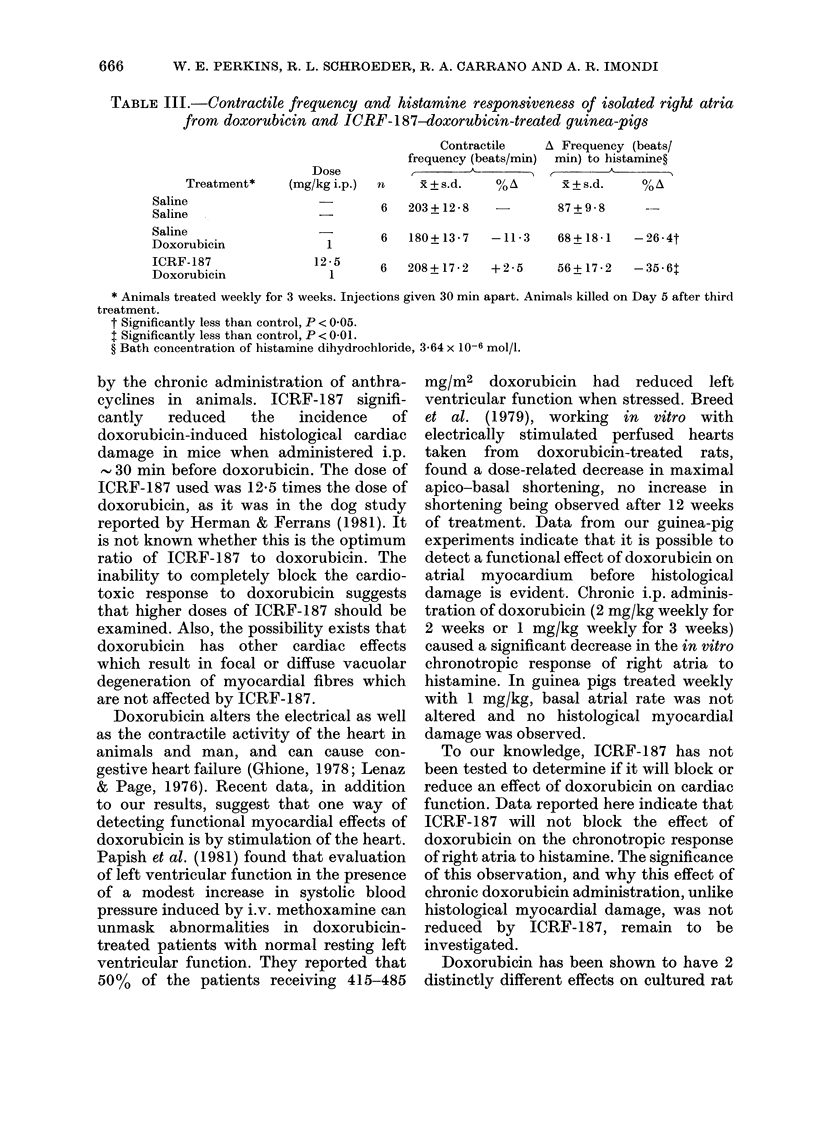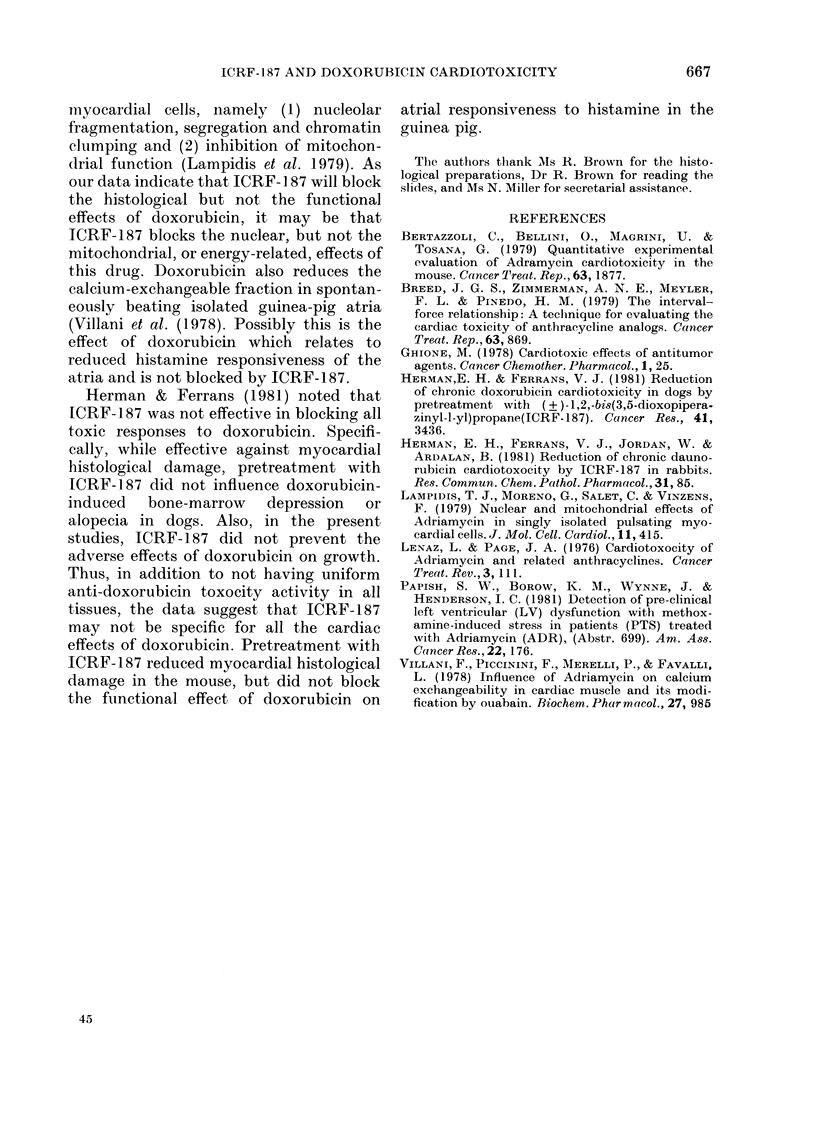# Effect of ICRF-187 on doxorubicin-induced myocardial effects in the mouse and guinea pig.

**DOI:** 10.1038/bjc.1982.251

**Published:** 1982-10

**Authors:** W. E. Perkins, R. L. Schroeder, R. A. Carrano, A. R. Imondi

## Abstract

ICRF-187 was tested for cardioprotective activity in doxorubicin-treated mice and guinea pigs. Pretreatment with i.p. ICRF-187 caused a significant decrease in the indicence of i.v. doxorubicin-induced myocardial histological damage in the mouse. I.p. ICRF-187 did not, however, reduce the effect of i.p. doxorubicin on a functional myocardial effect of this antitumour drug, a reduced histamine responsiveness of right atria in vitro. These data suggest that ICRF-187 may not be specific for all the cardiac effects of doxorubicin.


*Br. J. Cancer* (1982) **46**, 662

### EFFECT OF ICRF-187 ON DOXORUBICIN-INDUCED MYOCARDIAL EFFECTS IN THE MOUSE AND GUINEA PIG

W. E. PERKINS, R. L. SCHROEDER, R. A. CARRANO AND A. R. IMONDI

*From the Preclinical Research Division, Adria Laboratories Inc., P.O. Box 16529, Columbus, Ohio 43216, U.S.A.*

Received 18 March 1982 Accepted 17 June 1982

**Summary.—ICRF-187 was tested for cardioprotective activity in doxorubicin-treated mice and guinea pigs. Pretreatment with i.p. ICRF-187 caused a significant decrease in the incidence of i.v. doxorubicin-induced myocardial histological damage in the mouse. I.p. ICRF-187 did not, however, reduce the effect of i.p. doxorubicin on a functional myocardial effect of this antitumour drug, a reduced histamine responsiveness of right atria *in vitro*. These data suggest that ICRF-187 may not be specific for all the cardiac effects of doxorubicin.**

DOXORUBICIN (ADRIAMYCIN®) is an anthracycline antibiotic which has been playing a significant and increasingly important role in the treatment of cancer. However, because of a cardiomyopathy which it causes with increasing frequency at high cumulative doses, it has been necessary to limit the total amount of doxorubicin to 550 mg/m$^2$. The clinical utility of the agent would probably increase if this toxicity could be prevented or reduced without alteration of chemotherapeutic activity. Recently, Herman and colleagues have reported that ICRF-187, the (+)-isomer of ICRF-159 [(±)-1,-2-bis (3,5,dioxopiperazinyl-l-yl) propane], reduced cardiac damage in doxorubicin-treated dogs (Herman & Ferrans, 1981) and daunomycin-treated rabbits (Herman *et al.* 1981). The present studies were carried out to determine whether ICRF-187 affords any protection against the histological lesions caused by doxorubicin in the mouse heart. Studies were also conducted using guinea pigs to determine if ICRF-187 would reduce a functional effect of doxorubicin administration, namely a reduced chronotropic response of the right atrium to histamine *in vitro*.

### METHODS

*Mouse study.*—Female Cox ICR mice were obtained from Laboratory Animal Supply, Indianapolis, Ind., U.S.A. The animals were held in quarantine for 7 days and weighed 18–22 g at the start of the study. Purina Rodent Chow and water were permitted *ad libitum* throughout the study.

The experimental design was essentially as described by Bertazzoli *et al.* (1979) with the exception that the animals were injected i.p. with ICRF-187 (50 mg/kg) or saline (10 ml/kg) 30 min before receiving doxorubicin hydrochloride (4 mg/kg) or saline (10 ml/kg) i.v. Group 1 animals received saline i.p. and saline i.v., Group 2 saline i.p. and doxorubicin i.v., and Group 3 ICRF-187 i.p. and doxorubicin i.v. The animals were injected on Tuesday and Friday of Weeks 1, 2, 5, 6 and 7, and killed by ether asphyxiation during week 11 of the study. Hearts and kidneys were taken at necropsy and fixed in 10% buffered formalin. Routine paraffin sections (3 μm) of these organs were stained with haematoxylin and eosin and examined microscopically for damage. Severity of myocardial damage was graded as follows:

Grade 1—very slight; scattered, single myocardial fibres with vacuolation or degenerative changes.

Grade 2—slight; scattered small groups of

Correspondence to: William E. Perkins, Adria Laboratories Inc., Preclinical Research Division, P.O. Box 16529, Columbus, Ohio 43216, U.S.A.

altered myocardial fibres throughout the atrial and ventricular myocardium.

Grade 3—moderate; disseminated myocardial fibre vacuolation or degeneration with only occasional focal unaffected areas.

Grade 4—marked; confluent groups of affected myocardial fibres; most myocardial fibres affected.

The $\chi^2$ test was used to determine the significance of differences in lesion incidence among treatment groups.

*Guinea-pig studies.*—Male Hartley strain guinea pigs, (Davidson Mill Farm, Jamesburg, N.J., U.S.A.), weighing 258–369 g, were used in these experiments. The animals were caged individually with food and water permitted *ad libitum*. They were randomly divided into groups and after treatment, as defined below, were killed by stunning and exsanguination. The thoracic cavity was opened and the heart was rapidly extirpated and placed in a dish containing 95% $O_2$ and 5% $CO_2$-bubbled McEwan's solution (NaCl 7·60; KCl 0·42; $CaCl_2$, 0·24; $NaHCO_3$, 2·10; $NaH_2PO_4.H_2O$, 0·142; glucose 2·00; sucrose 4·50 g/l) at 33–35°C. The right atrium was isolated and suspended in a 10 ml isolated organ chamber containing McEwan's solution aerated with 95% $O_2$ and 5% $CO_2$ and maintained at 33–34°C. Atrial contraction was recorded using a Grass FT.03 force displacement transducer and a Beckman R612 Dynograph recorder. The atria were permitted to acclimatize for 30 min before addition of 0·1 ml of a $3{\cdot}64 \times 10^{-4}$ mol/l histamine dihydrochloride solution to each chamber to yield a bath concentration of $3{\cdot}64 \times 10^{-6}$ mol/l. This dose of histamine had previously been found to cause an increase in atrial rate of approximately 80% of maximum. Atrial rate was determined immediately before and 5 min after histamine treatment and the change in atrial rate was calculated for each preparation. Student's non-paired $t$ test was used to determine the significance of differences in basal rate and in the histamine response of atria from control and drug-treated animals.

*Expt 1: Effect of doxorubicin.*—One group of guinea pigs received a single i.p. injection of 1 mg/kg doxorubicin weekly on Days 1, 8 and 15 of the study; the second group received 2 mg/kg weekly, on Days 1 and 8. Control animals received saline (1 ml/kg) i.p. Animals from each group were killed 4 days after each injection of drug for the purpose of obtaining the atria. Hearts and kidneys from 2 control and 3 1mg/kg-doxorubicin-treated animals from the last group killed were collected and fixed in 10% buffered formalin. Routine paraffin sections (3 $\mu$m) of these organs, stained with haematoxylin and eosin, were examined microscopically.

*Expt 2: Effect of ICRF-187 and doxorubicin.*—Each guinea pig received 2 i.p. injections on Days 1, 8 and 15, either ICRF-187 (12·5 mg/kg) or saline (1 ml/kg) being injected 30 min before either doxorubicin (1 mg/kg) or saline (1 ml/kg). Group 1 animals received saline–saline, Group 2 saline–doxorubicin, and Group 3 ICRF-187–doxorubicin. The animals were observed and weighed daily and were killed on Day 20 of the study.

*Drugs*—ICRF-187 (NSC169780, Lot No. AD-01-81-1) was obtained from the National Cancer Institute, Silver Springs, MD, U.S.A. Histamine dihydrochloride was purchased from Sigma Chemical Co., St Louis, MO, U.S.A. Doxorubicin HCl was obtained from Farmitalia Carlo Erba, Milan, Italy.

### RESULTS

### *Mouse study*

Results of the histological evaluation of heart and kidney sections are summarized in Table I. Control animals had no histological evidence of myocardial damage. In contrast, focal myocardial damage was found in 8/15 (53%) mice treated with doxorubicin (4 mg/kg × 10). Six mice had Grade 1 (very slight) and 2 had Grade 2 (slight) myocardial damage. Focal mononuclear cellular infiltration was found in 2 of the 8 doxorubicin damaged hearts. ICRF-187 (50 mg/kg, i.p.), administered before each of the 10 doses of doxorubicin, significantly reduced the incidence of doxorubicin-induced myocardial damage ($P < 0{\cdot}05$). Only 3/17 (18%) of the mice treated with ICRF-187 and doxorubicin had Grade 1 myocardial lesions.

The incidence of microscopic renal damage was similar for each treatment group, being 10, 13, and 6% in the control, saline–doxorubicin, and ICRF-187–doxorubicin groups respectively. Mice in both the saline–doxorubicin ($28{\cdot}6 \pm 3{\cdot}4$ g; $\bar{x} \pm$ s.d.) and the ICRF-187–doxorubicin ($28{\cdot}1 \pm 2{\cdot}7$ g) groups weighed significantly less ($P < 0{\cdot}01$)

TABLE I.—*Histopathological effects of doxorubicin on hearts and kidneys of female mice*

| | Treatment* | | |
|---|---|---|---|
| Heart | Saline i.p. saline i.v. | Saline i.p. doxorubicin i.v. | ICRF-187 i.p. doxorubicin i.v. |
| No. examined | 10 | 15 | 17 |
| Incidence of cardiac damage | 0% | 53% | 18%† |
| No. hearts with‡ | | | |
| Myocardial vaculoation | | | |
| Grade 1 | 0 | 6 | 3 |
| Grade 2 | 0 | 2 | 0 |
| Grade 3 | 0 | 0 | 0 |
| Grade 4 | 0 | 0 | 0 |
| Focal mononuclear cellular infiltration | 0 | 2 | 0 |
| Kidneys | | | |
| No. examined | 10 | 15 | 17 |
| Incidence of renal damage | 10% | 13% | 6% |
| No. kidneys with: | | | |
| Focal mineralization, papilla | 1 | 0 | 1 |
| Tubular basophilia | 0 | 1 | 0 |
| Cystic tubule | 0 | 1 | 0 |

* Dosing schedule of Bertazzoli *et al.* (1979); twice weekly (Tuesday and Friday), Weeks 1, 2, 5, 6 and 7. I.p. injections were given 30 min before i.v. injections. The animals were killed during Week 11 of the study.
† Significantly less than saline–doxorubicin; $P < 0{\cdot}05$ ($\chi^2$ test).
‡ Refer to Methods for description of grades of myocardial vacuolation.

than the control (34·5 ± 5·4 g) animals at the end of the experiment. The only animal to die during the experiment, an animal in the ICRF-187–doxorubicin group, died 3 days after receiving the fourth pair of injections.

*Guinea-pig studies*

Results of an initial experiment to determine the effect of weekly i.p. administration of doxorubicin on the rate of contraction and response to histamine of atria mounted *in vitro* are summarized in Table II. Administration of 1, 2 or 3 doses of doxorubicin had no significant effect on the intrinsic rate of contraction of the atria. Significant reductions in the chronotropic response to histamine ($3{\cdot}64 \times 10^{-6}$ mol/l) were observed only after 2 weekly injections of 2 mg/kg (−30·9%) and 3 weekly injections of 1 mg/kg (−27·3%) doxorubicin. No evidence of vacuolar cardiomyopathy in the heart or degenerative or inflammatory changes in the cortex of the kidneys was found on histological examination of these tissues taken from 3 guinea pigs killed after the third 1mg/kg doxorubicin dose.

Results of the experiment to determine the effect of ICRF-187 on doxorubicin-induced functional effects on the atrium are shown in Table III. The administration of doxorubicin (1 mg/kg) once a week for 3 weeks did not affect atrial rate, but significantly reduced the chronotropic response of the atria to histamine by 26·4% (P < 0·05). ICRF-187 did not block this effect, the response of the atria taken from the ICRF-187 and doxorubicin-treated animals to histamine being reduced by 35·6% compared with control (P < 0·01). The control and ICRF-187–doxorubicin-treated animals gained spectively, 208 ± 21 ($\bar{x}$ ± s.d.) and 40 ± 53 g during the course of the study, while the saline–doxorubicin treated animals lost an average of 27 ± 28 g. Two animals in the saline–doxorubicin group died after receiving the third dose of doxorubicin.

### DISCUSSION

Data reported in this communication support published reports (Herman & Ferrans, 1981; Herman *et al.*, 1981) indicating that ICRF-187, the more water-soluble (+) isomer of ICRF-159, reduces myocardial histological damage induced

TABLE II.—*Contractile frequency and histamine responsiveness of isolated atria from guinea-pigs treated with doxorubicin*

| | | Contractile frequency (beats/min) | | | | | | Δ Frequency (beats/min) to histamine§ | | | | | |
|---|---|---|---|---|---|---|---|---|---|---|---|---|---|
| | | week 1† | | Week 2 | | Week 3 | | Week 1 | | Week 2 | | Week 3 | |
| Treatment* | Dose (mg/kg i.p.) | x̄±s.d. | %Δ | x̄±s.d. | %Δ | x̄±s.d. | %Δ | x̄±s.d. | %Δ | x̄±s.d. | %Δ | x̄±s.d. | %Δ |
| Saline | — | 186±15·9 (n=3) | — | 172·5±22·6 (n=4) | — | 199±16·7 (n=5) | — | 110±13·8 (n=3) | — | 102±16·2 (n=4) | — | 99·6±13·8 (n=5) | — |
| Doxorubicin | 1 | 204±22·4 (n=4) | +9·7 | 198±15·5 (n=4) | +14·8 | 194±15·6 (n=5) | −2·5 | 87±31·2 (n=4) | −20·9 | 94·5±13·3 (n=4) | −7·3 | 72·4±12·3 (n=5) | −27·3‡ |
| Doxorubicin | 2 | 181·5±27·0 (n=4) | −2·4 | 180±8·5 (n=4) | +4·3 | — | — | 99±29·6 (n=4) | −10·0 | 70·5±15·8 (n=4) | −30·9‡ | — | — |

* Animals injected weekly for a total of 1, 2 or 3 injections.

† Animals killed on Day 4 following injections 1, 2 and 3.

‡ Significantly less than control, $P < 0 \cdot 05$.

§ Bath concentration of histamine dihydrochloride, $3 \cdot 64 \times 10^{-6}$ mol/l.

TABLE III.—*Contractile frequency and histamine responsiveness of isolated right atria from doxorubicin and ICRF-*187*–doxorubicin-treated guinea-pigs*

| Treatment* | Dose (mg/kg i.p.) | $n$ | Contractile frequency (beats/min) x̄ ± s.d. | Contractile frequency (beats/min) %Δ | Δ Frequency (beats/min) to histamine§ x̄ ± s.d. | Δ Frequency (beats/min) to histamine§ %Δ |
|---|---|---|---|---|---|---|
| Saline<br>Saline | —<br>— | 6 | 203 ± 12·8 | — | 87 ± 9·8 | — |
| Saline<br>Doxorubicin | —<br>1 | 6 | 180 ± 13·7 | −11·3 | 68 ± 18·1 | −26·4† |
| ICRF-187<br>Doxorubicin | 12·5<br>1 | 6 | 208 ± 17·2 | +2·5 | 56 ± 17·2 | −35·6‡ |

* Animals treated weekly for 3 weeks. Injections given 30 min apart. Animals killed on Day 5 after third treatment.
† Significantly less than control, $P < 0{\cdot}05$.
‡ Significantly less than control, $P < 0{\cdot}01$.
§ Bath concentration of histamine dihydrochloride, $3{\cdot}64 \times 10^{-6}$ mol/l.

by the chronic administration of anthracyclines in animals. ICRF-187 significantly reduced the incidence of doxorubicin-induced histological cardiac damage in mice when administered i.p. ~30 min before doxorubicin. The dose of ICRF-187 used was 12·5 times the dose of doxorubicin, as it was in the dog study reported by Herman & Ferrans (1981). It is not known whether this is the optimum ratio of ICRF-187 to doxorubicin. The inability to completely block the cardiotoxic response to doxorubicin suggests that higher doses of ICRF-187 should be examined. Also, the possibility exists that doxorubicin has other cardiac effects which result in focal or diffuse vacuolar degeneration of myocardial fibres which are not affected by ICRF-187.

Doxorubicin alters the electrical as well as the contractile activity of the heart in animals and man, and can cause congestive heart failure (Ghione, 1978; Lenaz & Page, 1976). Recent data, in addition to our results, suggest that one way of detecting functional myocardial effects of doxorubicin is by stimulation of the heart. Papish *et al.* (1981) found that evaluation of left ventricular function in the presence of a modest increase in systolic blood pressure induced by i.v. methoxamine can unmask abnormalities in doxorubicin-treated patients with normal resting left ventricular function. They reported that 50% of the patients receiving 415–485 mg/m$^2$ doxorubicin had reduced left ventricular function when stressed. Breed *et al.* (1979), working *in vitro* with electrically stimulated perfused hearts taken from doxorubicin-treated rats, found a dose-related decrease in maximal apico–basal shortening, no increase in shortening being observed after 12 weeks of treatment. Data from our guinea-pig experiments indicate that it is possible to detect a functional effect of doxorubicin on atrial myocardium before histological damage is evident. Chronic i.p. administration of doxorubicin (2 mg/kg weekly for 2 weeks or 1 mg/kg weekly for 3 weeks) caused a significant decrease in the *in vitro* chronotropic response of right atria to histamine. In guinea pigs treated weekly with 1 mg/kg, basal atrial rate was not altered and no histological myocardial damage was observed.

To our knowledge, ICRF-187 has not been tested to determine if it will block or reduce an effect of doxorubicin on cardiac function. Data reported here indicate that ICRF-187 will not block the effect of doxorubicin on the chronotropic response of right atria to histamine. The significance of this observation, and why this effect of chronic doxorubicin administration, unlike histological myocardial damage, was not reduced by ICRF-187, remain to be investigated.

Doxorubicin has been shown to have 2 distinctly different effects on cultured rat myocardial cells, namely (1) nucleolar fragmentation, segregation and chromatin clumping and (2) inhibition of mitochondrial function (Lampidis *et al.* 1979). As our data indicate that ICRF-187 will block the histological but not the functional effects of doxorubicin, it may be that ICRF-187 blocks the nuclear, but not the mitochondrial, or energy-related, effects of this drug. Doxorubicin also reduces the calcium-exchangeable fraction in spontaneously beating isolated guinea-pig atria (Villani *et al.* (1978). Possibly this is the effect of doxorubicin which relates to reduced histamine responsiveness of the atria and is not blocked by ICRF-187.

Herman & Ferrans (1981) noted that ICRF-187 was not effective in blocking all toxic responses to doxorubicin. Specifically, while effective against myocardial histological damage, pretreatment with ICRF-187 did not influence doxorubicin-induced bone-marrow depression or alopecia in dogs. Also, in the present studies, ICRF-187 did not prevent the adverse effects of doxorubicin on growth. Thus, in addition to not having uniform anti-doxorubicin toxocity activity in all tissues, the data suggest that ICRF-187 may not be specific for all the cardiac effects of doxorubicin. Pretreatment with ICRF-187 reduced myocardial histological damage in the mouse, but did not block the functional effect of doxorubicin on atrial responsiveness to histamine in the guinea pig.

The authors thank Ms R. Brown for the histological preparations, Dr R. Brown for reading the slides, and Ms N. Miller for secretarial assistance.

### REFERENCES

BERTAZZOLI, C., BELLINI, O., MAGRINI, U. & TOSANA, G. (1979) Quantitative experimental evaluation of Adramycin cardiotoxicity in the mouse. *Cancer Treat. Rep.*, **63**, 1877.

BREED, J. G. S., ZIMMERMAN, A. N. E., MEYLER, F. L. & PINEDO, H. M. (1979) The interval–force relationship: A technique for evaluating the cardiac toxicity of anthracycline analogs. *Cancer Treat. Rep.*, **63**, 869.

GHIONE, M. (1978) Cardiotoxic effects of antitumor agents. *Cancer Chemother. Pharmacol.*, **1**, 25.

HERMAN, E. H. & FERRANS, V. J. (1981) Reduction of chronic doxorubicin cardiotoxicity in dogs by pretreatment with (±)-1,2,-*bis*(3,5-dioxopiperazinyl-l-yl)propane(ICRF-187). *Cancer Res.*, **41**, 3436.

HERMAN, E. H., FERRANS, V. J., JORDAN, W. & ARDALAN, B. (1981) Reduction of chronic daunorubicin cardiotoxocity by ICRF-187 in rabbits. *Res. Commun. Chem. Pathol. Pharmacol.*, **31**, 85.

LAMPIDIS, T. J., MORENO, G., SALET, C. & VINZENS, F. (1979) Nuclear and mitochondrial effects of Adriamycin in singly isolated pulsating myocardial cells. *J. Mol. Cell. Cardiol.*, **11**, 415.

LENAZ, L. & PAGE, J. A. (1976) Cardiotoxocity of Adriamycin and related anthracyclines. *Cancer Treat. Rev.*, **3**, 111.

PAPISH, S. W., BOROW, K. M., WYNNE, J. & HENDERSON, I. C. (1981) Detection of pre-clinical left ventricular (LV) dysfunction with methoxamine-induced stress in patients (PTS) treated with Adriamycin (ADR), (Abstr. 699). *Am. Ass. Cancer Res.*, **22**, 176.

VILLANI, F., PICCININI, F., MERELLI, P., & FAVALLI, L. (1978) Influence of Adriamycin on calcium exchangeability in cardiac muscle and its modification by ouabain. *Biochem. Pharmacol.*, **27**, 985